# Intrastriatal injection of alpha-synuclein preformed fibrils to rats results in L-DOPA reversible sensorimotor impairments and alterations in non-motor function

**DOI:** 10.3389/fnins.2025.1556447

**Published:** 2025-04-01

**Authors:** Sheila M. Fleming, Sophia Scott, Edward J. Hamad, Danielle E. Herman, John G. Holden, Lily Yan, Katrina Linning-Duffy, Christopher J. Kemp, Joseph R. Patterson, Kathryn M. Miller, Michael Kubik, Nathan Kuhn, Anna C. Stoll, Megan F. Duffy, Kathy Steece-Collier, Allyson Cole-Strauss, Jack W. Lipton, Kelvin C. Luk, Caryl E. Sortwell

**Affiliations:** ^1^Department of Pharmaceutical Sciences, Northeast Ohio Medical University, Rootstown, OH, United States; ^2^Department of Psychology, University of Cincinnati, Cincinnati, OH, United States; ^3^Department of Psychology, Michigan State University, East Lansing, MI, United States; ^4^Department of Translational Neuroscience, Michigan State University, Grand Rapids, MI, United States; ^5^Department of Pathology and Laboratory Medicine, University of Pennsylvania, Philadelphia, PA, United States

**Keywords:** Parkinson’s disease, cognitive function, dopamine, bradykinesia, nigrostriatal

## Abstract

**Introduction:**

The alpha-synuclein (*α*-syn) preformed fibril (PFF) model of Parkinson’s disease (PD) is widely used in rodents to understand the mechanisms contributing to progression of pathology and neurodegeneration in the disorder. While the time course of pathology in the *α*-syn PFF rat model has been well characterized, it has been more challenging to determine reliable and reproducible behavior impairments. This is mainly due to *α*-syn PFF injections resulting in a partial nigrostriatal lesion that make motor anomalies more subtle and difficult to detect, just as in patients with PD. In the present study we sought to examine the effect of increased striatal distribution and injection quantity of *α*-syn PFFs in rats on accumulation of phosphorylated *α*-syn inclusions, nigrostriatal degeneration, sensorimotor behavior, and nonmotor function related to PD.

**Methods:**

Male Fischer 344 rats were injected unilaterally in the striatum with a total of 24μg *α*-syn PFFs distributed into three sites, or an equal volume of phosphate buffered saline (PBS) as a control condition. Sensorimotor function was assessed using a battery of behavioral tests sensitive to varying degrees of nigrostriatal neurodegeneration. Non-motor testing included assays for olfaction, emotional reactivity, cognitive function, and sleep.

**Results:**

At six months post injection, *α*-syn PFF rats displayed significant movement and somatosensory asymmetries compared with control rats. Time to initiate a forelimb step and time to contact an adhesive stimulus on the forepaw took significantly longer with the contralateral limb compared with the ipsilateral limb in *α*-syn PFF rats. Further, hindlimb stepping in the cylinder was significantly reduced in *α*-syn PFF-injected rats compared with controls. Cognitive function was also affected in the *α*-syn PFF rats, with investigation time significantly decreased in an object recognition test. Levodopa reversibility was observed in the movement initiation and cylinder tests. Postmortem analysis revealed a 55% loss of nigral tyrosine hydroxylase immunoreactive neurons and a 63% reduction in striatal dopamine content in *α*-syn PFF-injected rats.

**Conclusion:**

Thus, using the present *α*-syn PFF surgical parameters, sufficient nigrostriatal degeneration can be achieved to manifest significant motor and non-motor deficits. These rat *α*-syn PFF surgical parameters will be important for preclinical assessment of novel diseasemodifying therapies.

## Introduction

Parkinson’s disease (PD) is classically characterized by loss of nigrostriatal dopamine (DA) neurons in the midbrain, formation of intraneuronal alpha-synuclein (*α*-syn) positive Lewy bodies and Lewy neurites, and development of cardinal motor symptoms that include bradykinesia, resting tremor, rigidity, and postural instability. At the time of diagnosis, which is typically based on motor symptoms, there is already substantial loss of nigrostriatal DA neurons (Fearnley and Lees, 1991; Ma et al., 1997; Greffard et al., 2006). In addition, there are a host of non-motor symptoms involving extranigral pathology that also reduce the quality of life for patients (e.g., cognitive impairments, neuropsychiatric dysfunction, autonomic alterations, olfaction deficits). While there is reasonable symptomatic treatment early in the disease for many of the motor symptoms using DA pharmacotherapy, there are currently few symptomatic treatments for the non-motor impairments and no available disease-modifying therapies. Thus, there are several key goals for PD research including (1) earlier detection, (2) identification of biomarkers that can track disease progression, (3) development of neuroprotective therapies, and (4) improved symptomatic treatments. Animal models of PD have been and still are an important tool in meeting these urgent unmet needs.

The *α*-syn preformed fibril (PFF) model of PD is currently widely used because it recapitulates multiple aspects of hallmark PD pathology (Luk et al., 2012a; Patterson et al., 2019a; Polinski, 2021). Injection of *α*-syn PFFs in the striatum induces endogenous *α*-syn pathology that propagates to anatomically connected regions (Luk et al., 2012a), including accumulation of phosphorylated *α*-syn inclusion pathology in the anterior olfactory nucleus, insular cortex, amygdala, and substantia nigra at 2 months post injection (Patterson et al., 2019a). Between 2 and 4 months after *α*-syn PFF injection, in addition to *α*-syn pathology, there is a loss of tyrosine hydroxylase immunoreactivity (THir) in the substantia nigra pars compacta (SNpc), in the absence of overt SNpc degeneration (Duffy et al., 2018; Patterson et al., 2019a). Then at 6 months, depending on the quantity of *α*-syn PFFs injected and number of deposition sites within the striatum, degeneration of SNpc neurons can range from 25–50% compared with controls (Luk et al., 2012a; Paumier et al., 2015; Abdelmotilib et al., 2017; Duffy et al., 2018; Patterson et al., 2019a; Thomsen et al., 2021; Tozzi et al., 2021). While the majority of studies use *α*-syn PFFs in mice, *α*-syn PFF rat models can provide certain advantages over mice, particularly in behavioral analysis, as the behavioral repertoire of the rat is more complex than that of the mouse (Whishaw et al., 2001). Characterization of behavioral impairments is critical in animal models in order to evaluate the potential for novel treatments to prevent, stop, slow, or reverse motor and non-motor symptoms of the disorder. *α*-syn PFF studies in rats show some deficits in motor, cranial motor, and cognitive function (Paumier et al., 2015; Patterson et al., 2019a; Thomsen et al., 2021; Tozzi et al., 2021; Marino et al., 2023). However, since nigrostriatal neurodegeneration hovers at ~50%, these deficits can be more variable and challenging to detect.

Decades ago, Schallert and colleagues developed numerous sensitive and reliable sensorimotor tests that correlate with striatal DA content and nigrostriatal degeneration in the unilateral 6-hydroxydopamine (6-OHDA) rat model of PD (Olsson et al., 1995; Kirik et al., 1998; Chang et al., 1999; Schallert and Tillerson, 2000). Initially these tests were used to study severe DA lesions (80–95% cell loss); however, as partial lesion models were developed many of these tests have proven sensitive in detecting more subtle sensorimotor impairments (Kirik et al., 1998; Fleming et al., 2005; Gombash et al., 2014). The tests showing sensitivity to 6-OHDA-induced partial lesions include adjusting step, movement initiation, limb-use asymmetry (cylinder test), and somoatosensory asymmetry (Dot) tests (Kirik et al., 1998; Fleming et al., 2005). In our previous work with the *α*-syn PFF rat, we found modest deficits in a modified version of the adjusting step test, more pronounced impairments in vocalizations, but no significant deficits in the limb-use asymmetry test, although in both studies there was a trend for decreased contralateral forelimb use by 6 months post injection (Paumier et al., 2015; Patterson et al., 2019a). Several recent studies in *α*-syn PFF-injected rats have also examined aspects of both motor and non-motor behavior. Thomsen et al. (2021) found a delayed approach in a corridor task but no differences in limb-use asymmetry; Tozzi et al. (2021) and Marino et al. (2023) showed an increased latency to climb an inclined grid, decreased time spent in the center in the open field, and spatial recognition deficits in rats with bilateral intrastriatal *α*-syn PFF injections. However, to date, there is no consensus on what motor and non-motor tests are optimal to use in the *α*-syn PFF rat.

The present study sought to expand upon previous research by using novel *α*-syn PFF injection parameters targeted to increase *α*-syn pathology in the cingulate cortex and nigrostriatal circuitry, with the intent to augment nigrostriatal degeneration, sensorimotor dysfunction, and non-motor function. Behavior was measured using a battery of well-established tests including movement initiation, spontaneous activity, postural stability, somatosensory asymmetry, cognitive function, emotional reactivity, sleep, and olfaction (Schallert et al., 1982; Schallert and Tillerson, 2000; Fleming et al., 2004, 2005, 2008; Walf and Frye, 2007; Brown et al., 2010; Mittal et al., 2020). L-DOPA responsiveness was also measured in movement initiation, somatosensory asymmetry, and spontaneous activity. Striatal DA content and stereological analysis of SNpc DA neurons were performed to determine the extent of nigrostriatal degeneration and their relationship with behavior impairments.

## Materials and methods

### Animals

**Figure 1:**
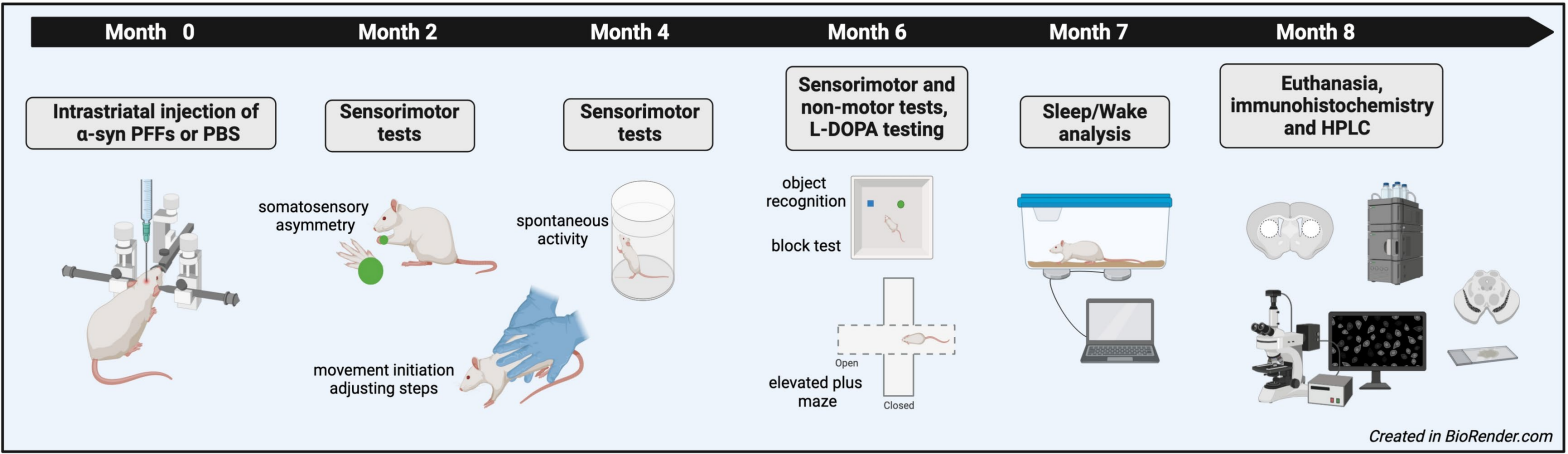
Schematic representation of the experimental design.

Three-month-old, male Fischer 344 rats (Total *n* = 19; PBS = 8, PFF = 11) were purchased from Charles River Laboratories. Group sizes were determined based on power analysis from previous studies using the behavioral tests included in the present experiments and are comparable to the initial *α*-syn PFF study in mice (Luk et al., 2012a; Mittal et al., 2020). Rats were initially housed at Michigan State University (MSU) with 1–2 per cage in a room with a 12 h light/dark cycle and provided food and water ad libitum. All rats received surgery at MSU and then 10 days later were shipped to Northeast Ohio Medical University (NEOMED) for behavioral testing. At NEOMED, rats were housed in the Comparative Medicine Unit with the same cage mate prior to shipping. The room was maintained on a reverse light/dark cycle to allow for behavior testing during the active period for rats. Rats remained at NEOMED for ~6.5 months for testing and then were shipped back to MSU for sleep analysis followed by euthanasia. All procedures were approved by MSU and NEOMED Institutional Animal Care and Use Committees. An overview of the experimental design is provided in Figure 1.

### *α*-syn PFF preparation and fibril measurements

*α*-syn PFFs were generated from wild-type-full length, recombinant mouse *α*-syn monomers as previously described (Polinski et al., 2018; Luk et al., 2012). Quality control was completed on full length fibrils to ensure fibril formation (transmission electron microscopy), amyloid structure (thioflavin T assay), pelletability as compared with monomers (sedimentation assay), and low endotoxin contamination (*Limulus* amebocyte lysate assay; <0.5 endotoxin units/mg of total protein). On surgery day, *α*-syn PFFs were thawed to room temperature and diluted to 4 μg/μl in sterile Dulbecco’s PBS and sonicated with an ultrasonic homogenizer (Q125 Sonicator; Qsonica, Newtown, CT); amplitude 30%; 60, 1 s pulses with 1 s between pulses. A sample of sonicated *α*-syn PFFs was prepared on Formvar/carbon-coated copper grids (EMSDIASUM, FCF300-Cu). Fibrils were then imaged with a JEOL JEM-1400+ transmission electron microscope (Patterson et al., 2019b). The length of ~650 fibrils was determined using ImageJ 1.53 K (Wayne Rasband and contributors, National Institutes of Health, United States). The mean fibril length was 47.19 ± 0.60 nm. Fibril length < 50 nm is required for efficient seeding of endogenous *α*-syn inclusions (Tarutani et al., 2016).

### Stereotaxic injections

Unilateral intrastriatal *α*-syn PFF injections were modified from previously described procedures. Specifically, a third injection site in the rostral striatum was added to the previous 2-site injection parameters (Patterson et al., 2019a) in order to increase *α*-syn pathology in the anterior cingulate cortex as well as to increase PFF seeding of the SNpc (Mailly et al., 2013). Rats were anesthetized with isoflurane (5% induction and 1.5% maintenance) and received three separate unilateral intrastriatal injections to the left hemisphere (3 × 2 μL, AP +2.0, ML +2.5, DV -4.5; AP +1.0, ML +2.0, DV -4.0; AP +0.1, ML +4.2, DV -5.0, AP and ML coordinates relative to Bregma, DV coordinates relative to dura). *α*-syn PFFs (4 μg/μl; 24 μg total) or an equal volume of PBS were injected at a rate of 0.5 μL/min with a pulled glass capillary tube attached to a 10 μL Hamilton syringe (Patterson et al., 2019b). To avoid *α*-syn PFF displacement, the needle was left in place for 1 min following injection, retracted 0.5 mm and left for 2 min before full retraction. All animals received analgesic (1.2 mg/kg of sustained release buprenorphine, s.c.) after surgery.

### Sensorimotor tests

#### Spontaneous activity

To measure limb use, rearing, and stepping, animals were placed in a clear plexiglass cylinder (17.6 cm inside diameter, 34 cm height) and activity was measured (Schallert et al., 2000; Schallert and Tillerson, 2000; Fleming et al., 2004, 2005). Animals with significant nigrostriatal damage have been shown to rear and step less and use the contralateral limb less for weight adjusting movements (Johnson et al., 1999; Schallert and Tillerson, 2000). Animals were videorecorded in the cylinder for five minutes and rears, steps, and limb use placement along the walls of the cylinder were measured. Videos were rated and analyzed by an experimenter blind to experimental condition. Animals were tested at 2, 4, and 6, months post-injection.

#### Movement initiation

To test movement initiation, stepping movements made by each forelimb were assessed with an isolated forelimb test (Schallert et al., 1992; Olsson et al., 1995; Schallert and Tillerson, 2000; Fleming et al., 2004, 2005). The rat was held by its torso with its hindlimbs and one forelimb lifted above the surface of a table so that the weight of the animal’s body was supported by one forelimb alone. The time to initiate one step was measured for each forelimb for three trials. The mean of the three trials for the contralateral and ipsilateral limbs was used to generate a Contralateral-Ipsilateral score (Contra-Ipsi Index; contralateral initiation time − ipsilateral initiation time/contralateral initiation time + ipsilateral initiation time) for each animal where a positive score represents a longer time to initiate with the contralateral limb. Animals were tested at 2, 4, and 6, months post-injection.

#### Adjusting steps

The adjusting step test is used to measure postural stability (Schallert and Tillerson, 2000; Fleming et al., 2004; Patterson et al., 2019a). In this test, animals were held in the same manner as in the movement initiation test where one forelimb bears the weight of the animal. The animal was then moved laterally across a distance of one meter on top of a cart. The number of adjusting steps made as the animal was moved across a table was recorded for each forelimb. The average number of steps in two trials for each forelimb was measured. Animals were tested at 2, 4, and 6, months post-injection.

#### Somatosensory asymmetry

Somatosensory asymmetry was measured using the “Dot” test (Schallert et al., 1982, 1983, 2000; Schallert and Whishaw, 1984; Schallert and Tillerson, 2000; Fleming et al., 2005). The test was performed by removing the animal from the home cage and attaching adhesive stimuli (Tough Spots adhesive-backed labels, 113 mm^2^) to the radial/ulnar aspect of each forepaw in random order. After being returned to the home cage, rats contact the stimuli one at a time. The order and latency of stimulus contact was measured for three trials. The mean of the three trials for the contralateral and ipsilateral forepaws was used to generate a Contralateral-Ipsilateral score (Contra-Ipsi Index; contralateral contact time − ipsilateral contact time/ contralateral contact time + ipsilateral contact time) for each animal where a positive score represents a longer contact or removal time with the contralateral limb. Animals were tested at 2, 4, and 6, months post-injection.

### Non-motor tests

Non-motor tests included assessment of emotional reactivity, cognition, olfaction, and sleep which are commonly affected in PD. Since many of these tests can be influenced by repeated testing, measurement occurred at only the 6 month timepoint. At 6 months there is established nigrostriatal degeneration, *α*-syn pathology in extranigral regions, and potentially a maximum effect on the non-motor behaviors (Patterson et al., 2019a).

#### Elevated plus maze

This test measures emotional reactivity in rodents (Walf and Frye, 2007). In this test animals were placed in the middle of a plastic maze that is the shape of a plus sign. Two of the arms/alleys have walls and two do not have walls (total alley length = 102 cm long, wall height = 30 cm, maze height = 50 cm). Animals were videorecorded for five minutes and time spent in each arm was measured by an experimenter blind to condition. The apparatus was thoroughly cleaned between animals. Animals were tested at 6 months post-injection.

#### Object recognition

Cognitive function was assessed using an object recognition test that measures attention and memory (Brown et al., 2010; Mittal et al., 2020). For this task each animal was placed in an opaque open field bin (length = 61 cm, width = 37 cm, height = 33 cm) and habituated for 20 min for 2 days prior to testing. On the day of the test, two trials were conducted, a Sample Trial and a Test Trial. For the Sample Trial, two identical objects were placed in one end of the bin on the same side. A rat was then placed in the bin and allowed to freely explore for 10 min. The rat was then put back in their home cage for 60 min. For the Test Trial, the rat was placed back in the apparatus, but this time one of the objects was replaced with a novel object. The rat was allowed to freely explore for 10 min and then placed back in its home cage. All trials were videorecorded for later analysis by an experimenter blind to condition. Time spent investigating each object and the number of rears were measured. Animals were tested at 6 months post-injection.

#### Block test of olfaction

This test measures olfactory discrimination (Tillerson et al., 2006; Fleming et al., 2008; Lehmkuhl et al., 2014). Individually housed animals were exposed to five wood blocks (2.5 cm^3^) placed inside each animal’s cage overnight. On the day of the test, animals were transferred into a separate testing area and the blocks were removed along with some of the cage bedding, both of which were placed in a plastic bag labeled with the animal’s identification. This allowed the blocks to maintain the scent of that animal. Water bottles, food and metal cage grids were removed and just the filtered cage lid remained on the cage. Animals were habituated to the room for one hour. For Trial 1, blocks 1–4, originally from the animal’s own cage, were placed at the same time in the middle of the cage. The blocks were placed approximately 1–2 cm apart. The rat was then videorecorded for 30 s. All rats received a total of six exposures to blocks 1–4 with an inter-trial interval of ~5 min. On the seventh trial, block 4 was removed and replaced with block 5 from another rat’s bedding. Blocks 1–3 and 5 were then placed into the cage and the animal was videorecorded for 30 s as in Trials 1–6. The order of the blocks varied between rats. The experimenter wore gloves throughout the entire procedure and changed them for each trial for each rat. A rater, blind to experimental condition, measured the time spent sniffing each block, with sniffing defined as nasal contact with that block. Animals were tested at 6 months post-injection.

#### Sleep/wake monitoring and analysis

At ~7 months post-injection rats were singly housed in plexiglass cages (37 × 22 × 19cm) with a piezoelectric sensor pad placed underneath to detect pressure changes on the cage floor (Signal Solutions, Lexington, KY). This detection method is noninvasive and has been shown to achieve >90% accuracy for sleep/wake classification based on validation with simultaneous recording through EEG/EMG (Mang et al., 2014). After 1 week of habituation, sleep/wake was monitored over 2 weeks. Data acquisition continued in between weekly cage change, thus a total of 12 days (6 days per week) of recording was obtained for each animal, during which animals were left undisturbed. The acquired signals were converted and analyzed using SleepStats (Signal Solutions), as previously described (Donohue et al., 2008; Mang et al., 2014; Yaghouby et al., 2016). Amount (%) of sleep and average sleep bout length during day and night were determined for each day and averaged across the 12 days of recording period for each animal, then compared between groups.

### Levodopa

Following the 6 month emotional reactivity, cognitive, and olfaction testing and prior to sleep analysis, PBS and PFF rats were administered saline or Benserazide/L-DOPA over two sessions 1 week apart in a counterbalanced manner to determine deficit reversibility in the movement initiation, somatosensory asymmetry, and spontaneous activity tests. Animals were administered either saline or Benserazide (12.5 mg/kg, ip; B7283 Sigma-Aldrich) and then 20 min later they were administered saline or L-DOPA (25 mg/kg, ip; D1507 Sigma-Aldrich). Ten minutes after the second injection, movement initiation was performed followed by somatosensory asymmetry, and then spontaneous activity (Fleming et al., 2005, 2006; Hwang et al., 2005).

### Euthanasia

Rats were euthanized at ~8 months post-surgery. Rats were given a 30 mg/kg pentobarbital injection (i.p.; Euthanasia-III Solution, MED-PHARMEX, Inc.) and perfused intracardially with heparinized 0.9% saline. Brains were removed, hemisected in the coronal plane at ~ Bregma −2.5 mm. The rostral portion of the brain was immediately flash frozen in 2-methylbutane on dry ice for 10–20 s and subsequently stored at −80°C. The caudal portion of the brain was post-fixed in 4% paraformaldehyde (PFA) for 1 week and then transferred to 30% sucrose in 0.1 M phosphate buffer until sinking. Brains were frozen on dry ice and cut at 40 μm on a sliding microtome, sections were stored in cryoprotectant (30% sucrose, 30% ethylene glycol, in 0.1 M Phosphate Buffer (PB), pH 7.3) at −20°C.

### Immunohistochemistry

Free floating sections were washed 4 × 5 min in 0.1 M tris buffered saline (TBS) containing 0.5% Triton-X100 (TBS-Tx), quenched in 3% H_2_O_2_ for 1 h, blocked in 10% normal goat serum (NGS) in TBX-Tx, and incubated overnight in primary antibody in 1% NGS/TBS-Tx at 4°C on a shaker. Primary antibodies used included: mouse anti-*α*-syn phosphorylated at serine 129 (pSyn) (1:10,000; Abcam, AB184674), mouse anti-tyrosine hydroxylase (TH) (1:4000; Millipore, MAB318) and mouse anti Hu antigen C (HuC) (1:2000; Invitrogen, A-21271). Sections were washed in TBS-Tx and then incubated for 2 h at room temperature with biotinylated goat anti-mouse IgG (1:500; Millipore, AP124B) in 1% NGS/TBS-Tx. Sections were washed 4 × 5 min in TBS-Tx and incubated in standard avidin-biotin complex detection kit (ABC, Vector Laboratories, PK-6100). Immunolabeling was visualized with 0.5 mg/mL diaminobenzidine (Sigma-Aldrich, D5637), and 0.03% H_2_O_2_ in TBS-Tx. Sections were mounted, allowed to dry, rehydrated, then dehydrated in ascending ethanol washes and cleared with xylene before cover slipping using Epredia Cytoseal-60 (Thermo-Fisher, 22-050-262). pSyn sections were counterstained with cresyl violet before dehydration.

### Stereological assessment of SNpc TH and HuC Immunoreactive neurons

The number of TH immunoreactive (THir) and HuCir neurons in the ipsilateral and contralateral SNpc was estimated using unbiased stereology with the optical fractionator principle. The investigator was blinded to treatment groups. Using a Nikon Eclipse 80i microscope, Retiga 4000R camera (QImaging) and Microbrightfield StereoInvestigator software (Microbrightfield Bioscience, Williston, VT), quantification was completed by drawing a contour around the SNpc borders using the 4X objective on every sixth section and counting neurons according to stereological principles at 60X magnification. Briefly, counting frames (50 μm x 50 μm) were systematically and randomly distributed over a grid (273 μm x 119 μm for TH; 400 μm x 200 μm for HuC) overlaid on the SNpc. A coefficient of error < 0.10 was accepted. The total estimate of THir or HuCir neurons was calculated for each hemisphere, data are reported as percent of contralateral (uninjected) hemisphere.

### Quantification of monoamine levels in the striatum and cortex

Frozen brains were mounted in OTC and sectioned on a cryostat to the level of the rostral striatum (~ Bregma +2.2 mm). The cortex and striatum from both hemispheres were individually collected using a 2 mm x 2 mm tissue punch and stored at −80°C. Tissue was homogenized and analyzed as described previously (Koprich et al., 2003). The Pierce BCA Protein Kit (Rockford, IL) was utilized for protein determination. Samples were separated on a Microsorb MV C-18 column (5 Am, 4.6–250 mm, Varian, Palo Alto, CA) and simultaneously examined for dopamine (DA), homovanillic acid (HVA), 3,4-dihydroxyphenylacetic acid (DOPAC), norepinephrine (NE), 3-methoxy-4-hydroxyphenylglycol (MHPG), serotonin (5HT) and 5-hydroxyindoleacetic acid (5-HIAA). Compounds were detected using a 12-channel coulometric array detector (CoulArray 5,200, ESA, Chelmsford, MA) attached to a Waters 2,695 Solvent Delivery System (Waters, Milford, MA) under the following conditions: flow rate of 1 mL/min; detection potentials of 50, 175, 350, 400 and 525 mV; and scrubbing potential of 650 mV. The mobile phase consisted of a 10% methanol solution in distilled H_2_O containing 21 g/L (0.1 M) citric acid,10.65 g/L (0.075 M) Na2HPO4, 176 mg/L (0.8 M) heptanesulfonic acid and 36 mg/L (0.097 mM) EDTA at a pH of 4.1. Data are expressed as ng/mg protein.

### Statistics

All statistical tests were completed using GraphPad Prism software (version 10, GraphPad, La Jolla, CA) or MATLAB 2021A, Mathworks, Inc. Parametric and nonparametric statistics were used to compare control PBS and PFF rats. Since the PFF manipulation is unidirectional (i.e., the intervention is hypothesized to impair performance in the behavioral tests), all contrasts were performed using a one-tailed rejection region with the type one error rate set at 0.05 (Hays, 1994). Parametric analyses of somatosensory asymmetry and adjusting steps were performed using a 2 × 3 mixed design ANOVA to compare group (PBS and PFF) and time (2,4, and 6 m) followed by Tukey *post hoc* comparisons. Student’s *t*-test were used for comparisons between control PBS and PFF rats for percent of contralateral nigral THir neurons, HuCir neurons, and monoamine levels. Nonparametric statistics including Friedman’s ANOVA, Mann–Whitney U, and Wilcoxon Sign Rank were used to compare between factors and repeated measures and were performed on movement initiation, spontaneous activity, block test, object recognition, and elevated plus maze data. Paired Student’s *t*-test were used for analysis of saline and L-DOPA treatment on sensorimotor function. A two-way ANOVA followed by Tukey *post hoc* comparisons were made between percent of sleep and average sleep bout length with group (PBS and PFF) and time (day and night) as independent variables. Pearson correlations were performed between percent lesion of nigral THir neurons and percent lesion of striatal DA, as well as between behavior tests and percent lesion of striatal DA. Analysis of effect size using Cohen’s d and Pearson r were also performed on significant outcomes.

## Results

### *α*-syn PFF injections result in sensorimotor impairments at 6 months

**Figure 2:**
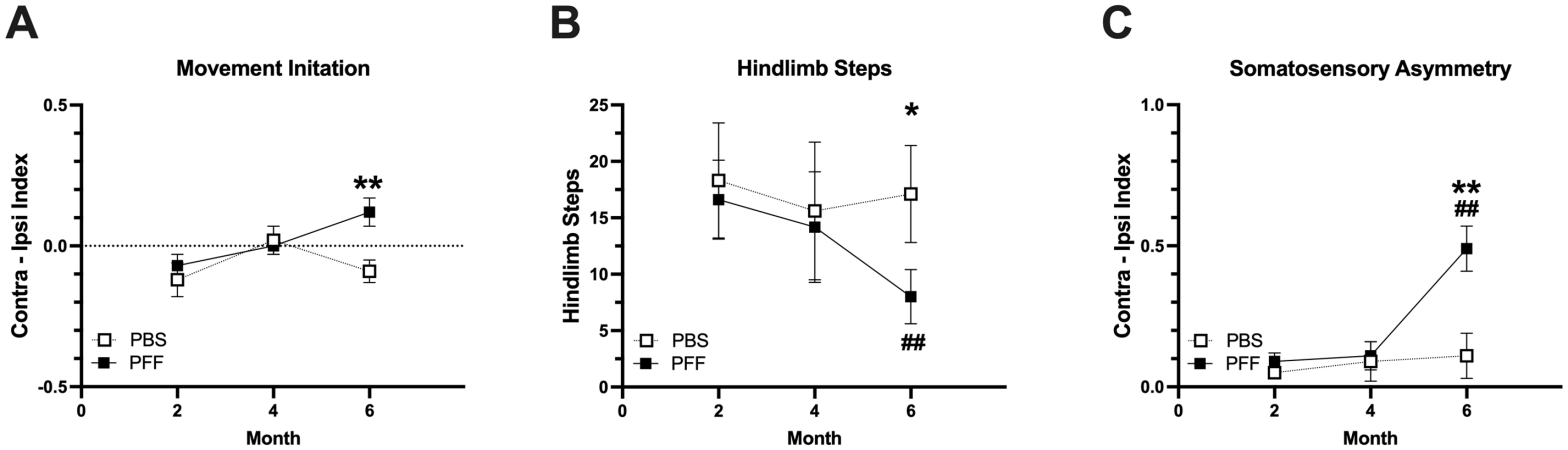
Sensorimotor deficits in *α*-syn PFF-injected rats. Sensorimotor assessment in intrastriatal PBS (*n* = 8) and PFF (*n* = 11) injected rats at 2, 4 and 6 months following surgery. **(A)** Bradykinesia was measured using the movement initiation test. Time to initiate a step was measured for the ipsilateral (Ipsi) and contralateral (Contra) forelimbs. The mean of three trials for contralateral initiation and ipsilateral initiation times (Contra-Ipsi Index) is presented. **(B)** Hindlimb stepping was measured using the cylinder test. **(C)** Sensorimotor asymmetry was measured using an adhesive removal test. Time to contact an adhesive stimulus was measured for the ipsilateral and contralateral forepaws for three trials. The mean contralateral and ipsilateral contact times were used to create a Contra-Ipsi Index (contralateral contact time − ipsilateral contact time/contralateral + ipsilateral contact time). *, ** represents *p* < 0.05, 0.01, respectively PBS vs. PFF. ## Represents *p* < 0.01 compared to 2-month PFF.

For movement initiation (Figure 2A), there was a significant difference between PBS and *α*-syn PFF injected rats at 6 months post injection in which *α*-syn PFF rats had higher Contra-Ipsi scores compared with controls (Mann Whitney U, *p* < 0.01) indicating PFF rats took longer to initiate a step with the contralateral limb compared to PBS rats. Although the PBS group had an overall negative mean Contra-Ipsi score (indicating slower initiation time for the ipsilateral limb), the scores were split with 3 rats initiating faster with the ipsilateral limb and 5 rats initiating slower with the ipsilateral limb. In contrast in the *α*-syn PFF group 9 out of 11 rats were slower to initiate a step with the contralateral limb. Friedman’s ANOVA showed no effect of repeated measures for PBS or *α*-syn PFF rats in movement initiation. For hindlimb steps in the cylinder (Figure 2B), there was a significant difference between PBS and *α*-syn PFF injected rats at 6 months post injection with *α*-syn PFF rats making fewer hindlimb steps compared with controls (Mann Whitney U, *p* < 0.05). In addition, 6 m *α*-syn PFF rats made significantly fewer hindlimb steps compared to the 2 m PFF timepoint (Wilcoxon Sign Rank, *p* < 0.01). For somatosensory asymmetry (Figure 2C), a 2 × 3 mixed ANOVA revealed a significant main effect of group (*F*[2, 17] = 7.55, *p* < 0.05), time (*F*[2, 34] = 8.86, *p* < 0.01), and a group X time interaction (*F*[2, 34] = 5.70, *p* < 0.01). Tukey’s post hoc showed that the 6 m *α*-syn PFF rats had significantly higher Contra-Ipsi scores compared to 6 m PBS rats and compared to the 2 m *α*-syn PFF timepoint (*p* < 0.01 for both). For adjusting steps (Table 1), a 2 × 3 mixed ANOVA revealed a significant main effect of time (*F*[2, 34] = 6.36, *p* < 0.01). Tukey’s post hoc showed 6 m PBS rats made fewer contralateral steps compared to the 2 m timepoint (*p* < 0.05).

**Table 1:** Additional outcome measures.

	Time post injection (months)		
Body weight (g)	2	4	6
PBS	314.5 ± 7.9	374.5 ± 12.2	413.8 ± 11.2
*α*-syn PFF	301.8 ± 4.6	358.6 ± 5.5	389.5 ± 5.9
Spontaneous activity			
*Rears*			
PBS	6.1 ± 0.7	4.21 ± 0.9	3.8 ± 1.8
*α*-syn PFF	4.5 ± 1.0	4.32 ± 1.6	3.4 ± 1.8
*Forelimb steps*			
PBS	25.2 ± 4.7	20.1 ± 5.6	14.1 ± 3.6
*α*-syn PFF	27.3 ± 6.2	26.4 ± 7.2	28.9 ± 6.7
Adjusting steps			
*Contralateral limb*			
PBS	9.6 ± 0.4	9.1 ± 0.7	7.1 ± 0.3 **Δ**
*α*-syn PFF	9.8 ± 0.4	9.4 ± 0.9	8.5 ± 0.3

Mean ± SEM, Δ *p* < 0.05 compared to PBS 2 m timepoint.

### PFF injections result in deficits in object recognition

**Figure 3:**
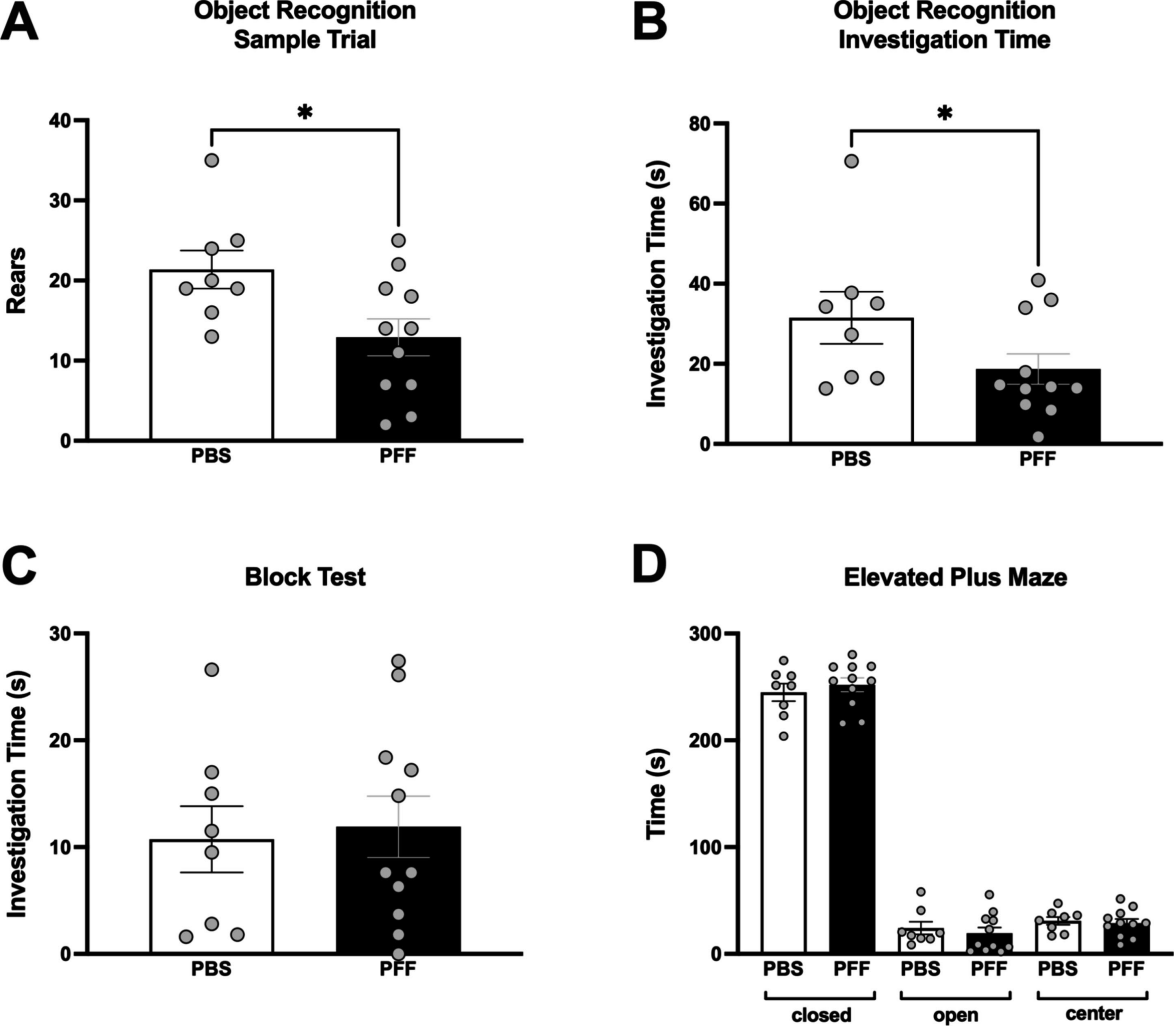
Deficits in object recognition in *α*-syn PFF-injected rats. Non-motor assessments in intrastriatal PBS (*n* = 8) and PFF (*n* = 11) injected rats at 6 months following surgery. Cognitive function was assessed using an object recognition test that measures attention and memory with **(A)** Rears in the sample trial and **(B)** total investigation time in the test trial shown. **(C)** Assessment of olfactory discrimination in the block test and **(D)** Assessment of emotional reactivity in the elevated plus maze. * Represents *p* < 0.05.

Non-motor testing was also performed at 6 months post injection. Significant differences between PBS and *α*-syn PFF rats were detected in aspects of the object recognition test. In the Sample Trial, *α*-syn PFF rats reared less than controls (Mann–Whitney U, *p* < 0.05, Figure 3A) and in the Test Trial, total investigation time was significantly decreased in *α*-syn PFF rats compared with controls (Mann–Whitney U, *p* < 0.05; Figure 3B). There were no significant differences between PBS and *α*-syn PFF rats in the elevated plus maze or block test (*p* > 0.05, Figures 3C,D).

### *α*-syn PFF-induced motor deficits are reversible with levodopa

**Figure 4:**
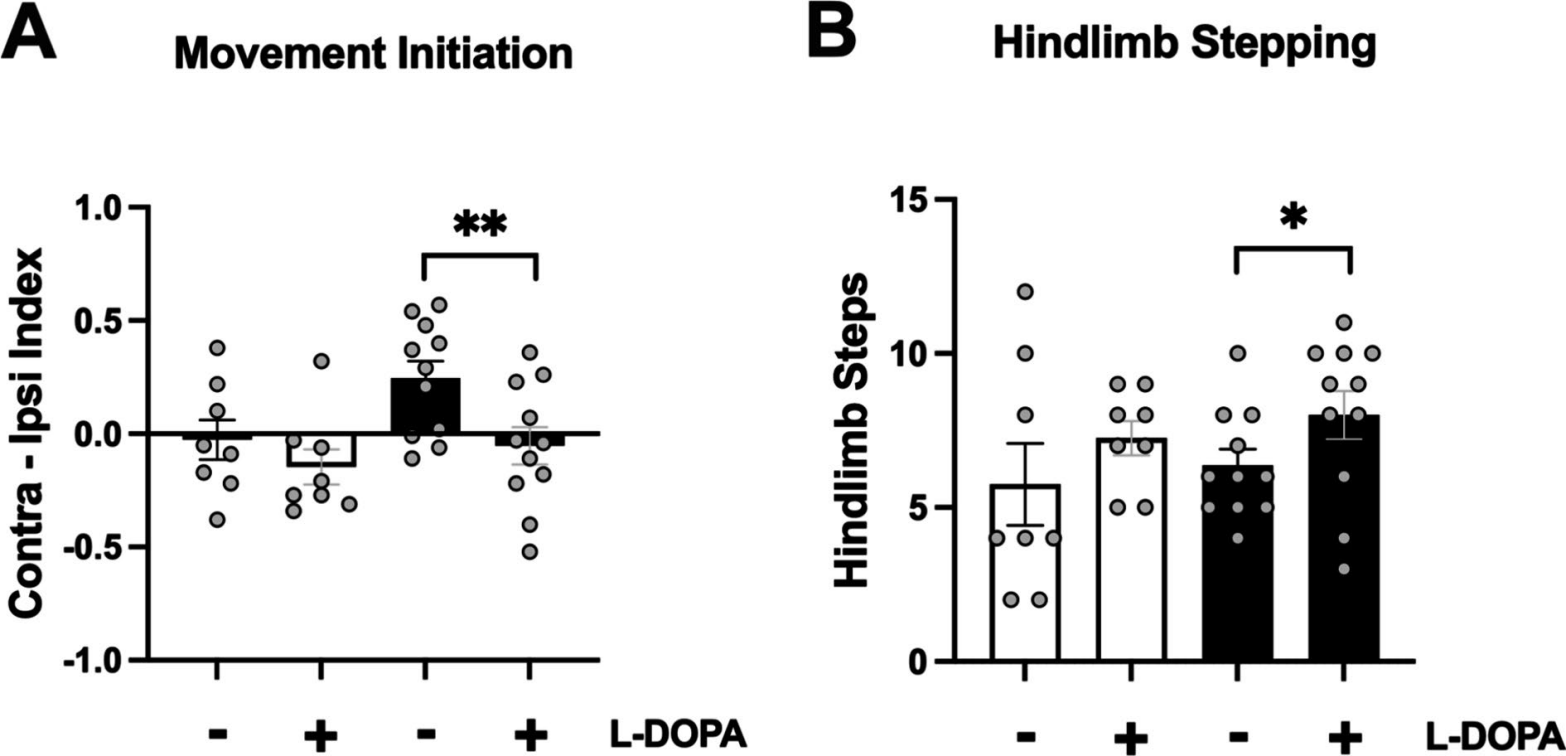
Effect of L-DOPA on *α*-syn PFF-induced impairments in sensorimotor function. Sensorimotor function was assessed in response to saline and L-DOPA (25 mg/kg) in PBS (*n* = 8, white bars) and *α*-syn PFF (*n* = 11, black bars) rats. **(A)** Movement initiation was improved with L-DOPA in *α*-syn PFF rats. The mean of three trials for contralateral initiation and ipsilateral initiation times were used to generate a Contra-Ipsi Index (contralateral initiation time – ipsilateral initiation time/contralateral initiation time + ipsilateral initiation time) for each animal. **(B)** Hindlimb stepping in the cylinder was improved with L-DOPA in *α*-syn PFF rats. Paired student *t*-test; *, ** represents *p* < 0.05, 0.01, respectively.

In the PBS rats there was no effect of either saline or L-DOPA administration on sensorimotor behavior (*p* > 0.05). However, in *α*-syn PFF rats, treatment with L-DOPA resulted in a significant reversal of deficits in movement initiation and hindlimb stepping. Specifically, L-DOPA-treated *α*-syn PFF rats showed significantly reduced asymmetry in movement initiation compared to rats given saline (Figure 4A; t(10) = 3.66, *p* < 0.01). There was also a modest reversal in hindlimb stepping in the cylinder, with L-DOPA-treated *α*-syn PFF rats making more hindlimb steps compared to their performance following a saline injection (Figure 4B; t(10) = 1.94, *p* < 0.05). L-DOPA did not affect adhesive removal in *α*-syn PFF rats (*p* > 0.05).

### Intrastriatal *α*-syn PFF injection is not associated with sleep deficits

**Figure 5:**
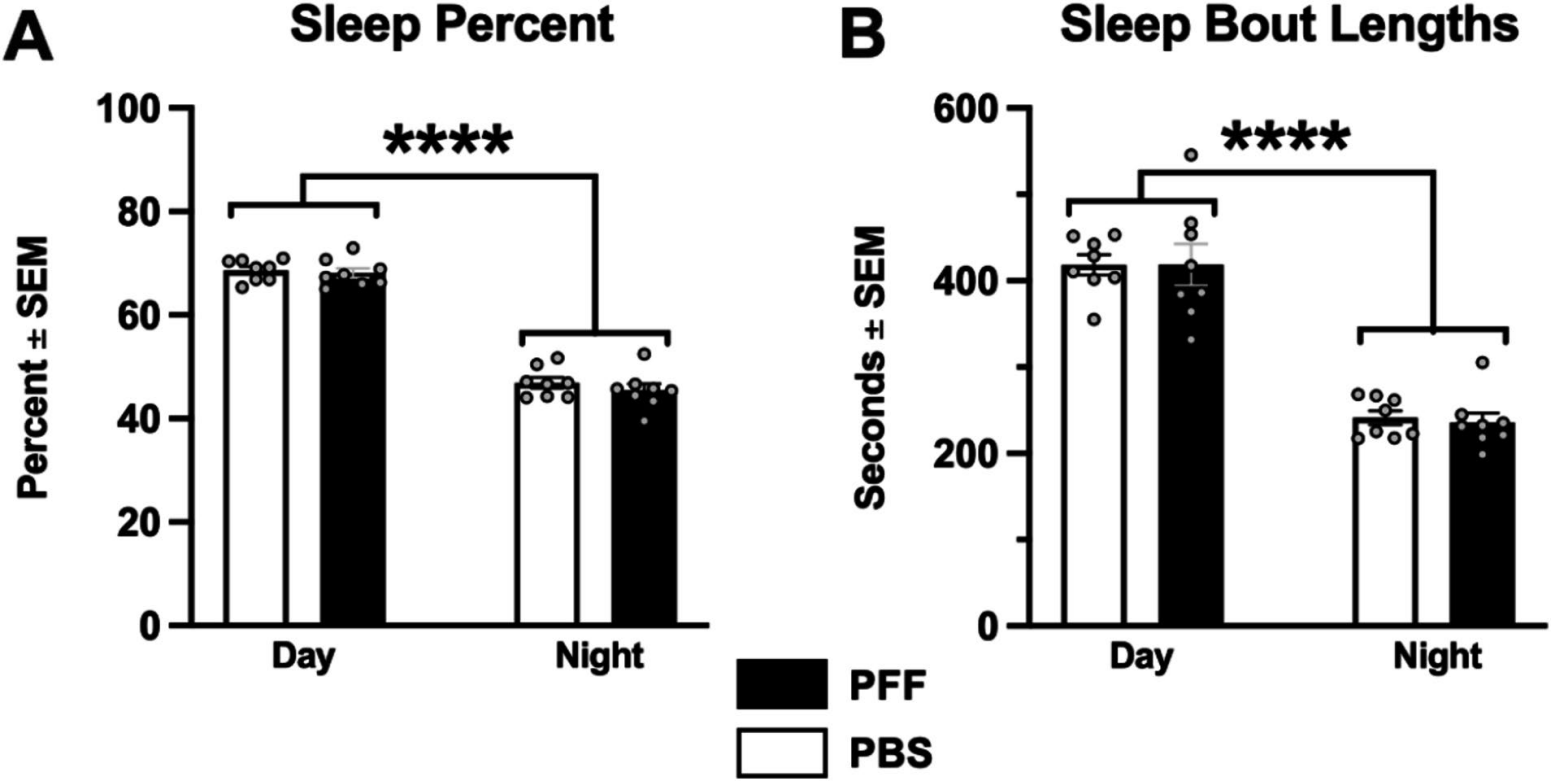
*α*-syn PFF injections not associated with sleep deficits. Sleep behavior was measured in intrastriatal PBS (*n* = 8) and *α*-syn PFF (*n* = 11) rats. **(A)** Percent time in sleep during the light and dark cycles and **(B)** sleep bout length did not differ between PBS control and *α*-syn PFF rats. 2 × 2 mixed design ANOVA, Tukey’s *post hoc*; **** represents *p* < 0.001.

A clear daily rhythm (day/night difference) in sleep was observed in both PBS and *α*-syn PFF rats. In both treatment groups, the percentage of sleep and length of sleep bouts were significantly higher during the day compared with night (Figures 5A,B; *p* < 0.0001). However, no differences in sleep percentage or sleep bout length were observed in *α*-syn PFF-injected rats (p > 0.05).

### *α*-syn PFF injections trigger pSyn accumulation and nigrostriatal degeneration

**Figure 6:**
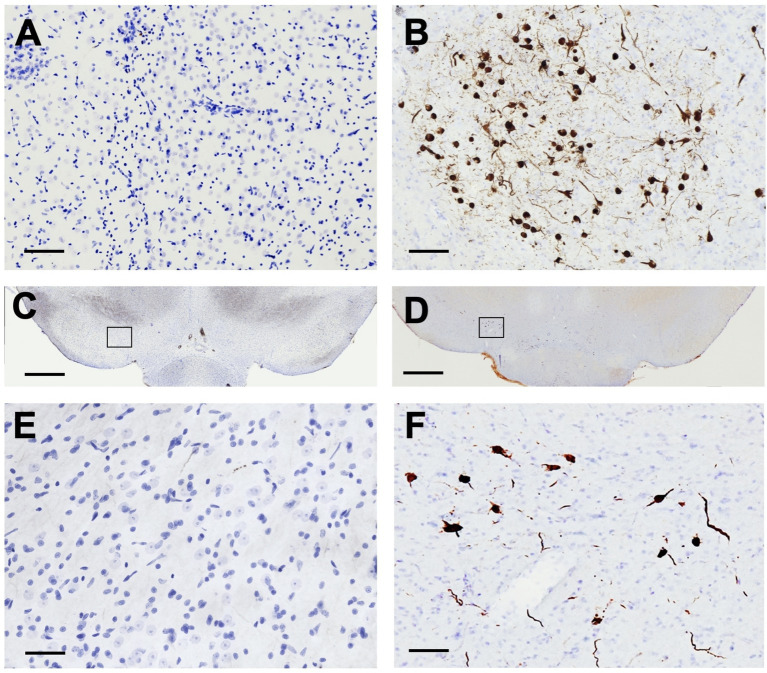
Intrastriatal *α*-syn PFF Injection results in accumulation of phosphorylated *α*-Syn. Sections were immunolabeled for phosphorylated *α*-Syn (pSyn) and counterstained with cresyl violet. pSyn accumulation was only detected in *α*-syn PFF rats. **(A,C,E)** No pSyn was observed within the amygdala **(A)** or the SNpc (**C**, with inset in **E**) in PBS rats 8 months past surgery. In contrast, in *α*-syn PFF-injected rats **(B,D,F)**, accumulation of pSyn was observed within the amygdala **(B)** and SNpc neurons (**D**, with inset in **F**) ipsilateral to the intrastriatal injection 8 months past surgery. Scale bar in **A,B** = 100 μM, scale bar in C,D = 1,000 μM, scale bar in **E,F** = 50 μM.

**Figure 7:**
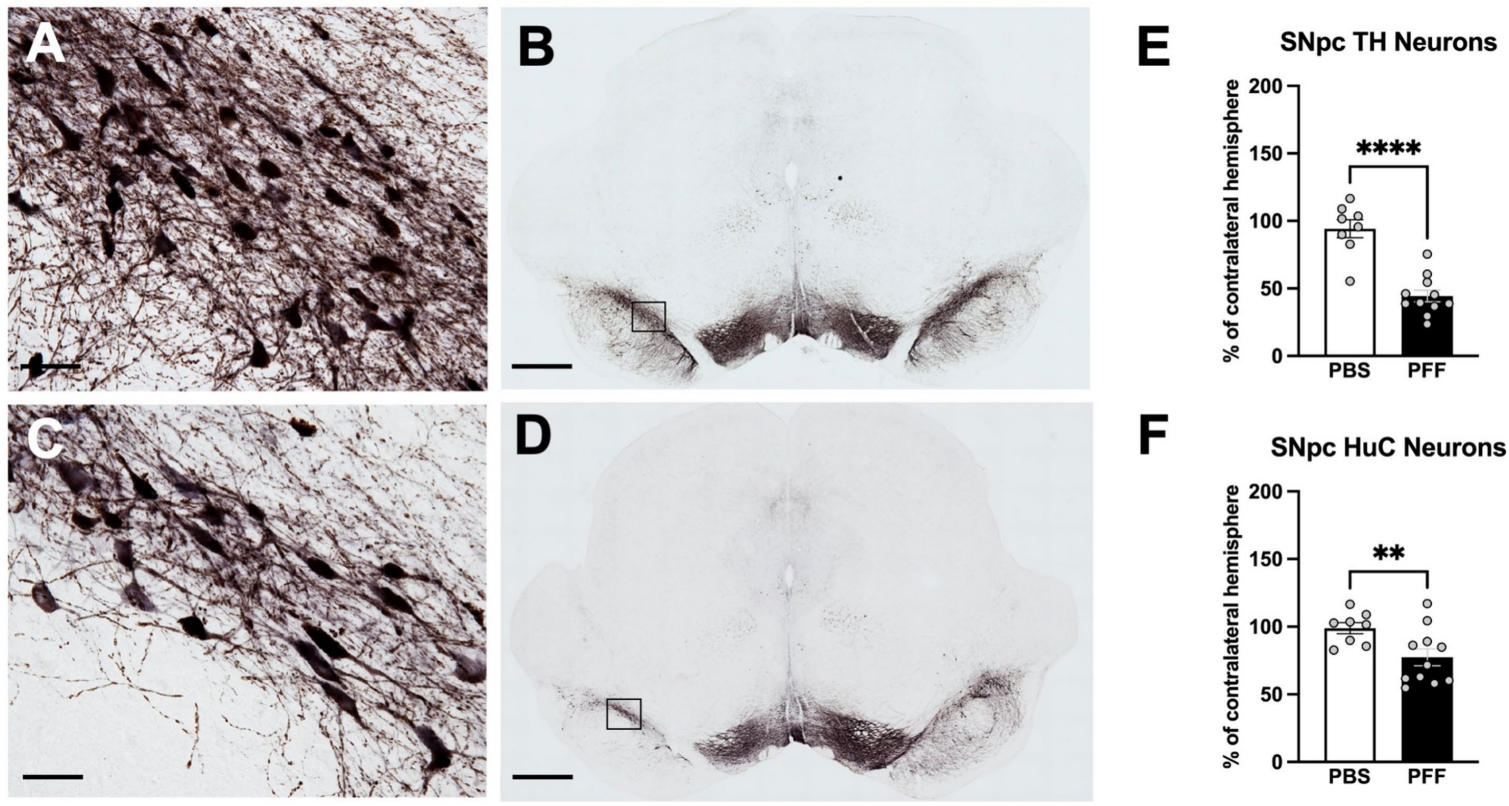
Intrastriatal *α*-syn PFF injection results in degeneration of SNpc neurons. Immunohistochemistry for tyrosine hydroxylase (TH) and Hu antigen C (HuC) in the SNpc was combined stereological assessment to quantify TH neurons and total neurons, respectively, in PBS and *α*-syn PFF rats 8 months following surgery. THir neurons in the SNpc in PBS **(A,B)** and PFF **(C,D)** injected rats. Stereological quantification of **(E)** THir neurons and **(F)** HuC neurons. Scale bar in A, C = 25 μM, scale bar in B, D = 1,000 μM. Student’s *t*-test; **, **** represents *p* < 0.01, 0.0001, respectively.

pSyn immunoreactivity (pSynir) was not observed in PBS control rats in any structure whereas in *α*-syn PFF injected rats, accumulation of pSyn was observed within the amygdala and SNpc ipsilateral to intrastriatal injection (Figures 6A–E). Intrastriatal injection of a lower quantity of PFFs, at 2 injection sites instead of the 3 used in the present study, demonstrates that pSyn accumulation in the ipsilateral SNpc peaks at 2 months and declines more than 80% from the peak between month 2 and month 6 (Duffy et al., 2018; Patterson et al., 2019a; Stoll et al., 2024a,b). Thus, the modest number of pSynir SNpc neurons observed in the SNpc in the present study is in line with expectations 8 months following *α*-syn PFF injection (Figures 6B,D,F). The decline in pSynir nigral neurons likely reflects degeneration of nigral neurons that were initially seeded by *α*-syn PFFs, as previously reported (Osterberg et al., 2015). Indeed, *α*-syn PFF rats displayed a 55% reduction in nigral THir neurons compared with control injected rats (Figure 7E; t(17) = 6.482, *p* < 0.0001). Further, quantification of HuCir SNpc neurons revealed a significant reduction in neurons in the ipsilateral SNpc of *α*-syn PFF injected rats (Figure 7F; t(17) = 2.645, *p* < 0.01). These results suggest that the loss of THir neurons associated with *α*-syn PFF injection reflects neuronal loss and not simply downregulation of TH phenotype (Figures 7A–F).

### *α*-syn PFF injections result in decreased striatal DA and metabolites and increased DA turnover

**Figure 8:**
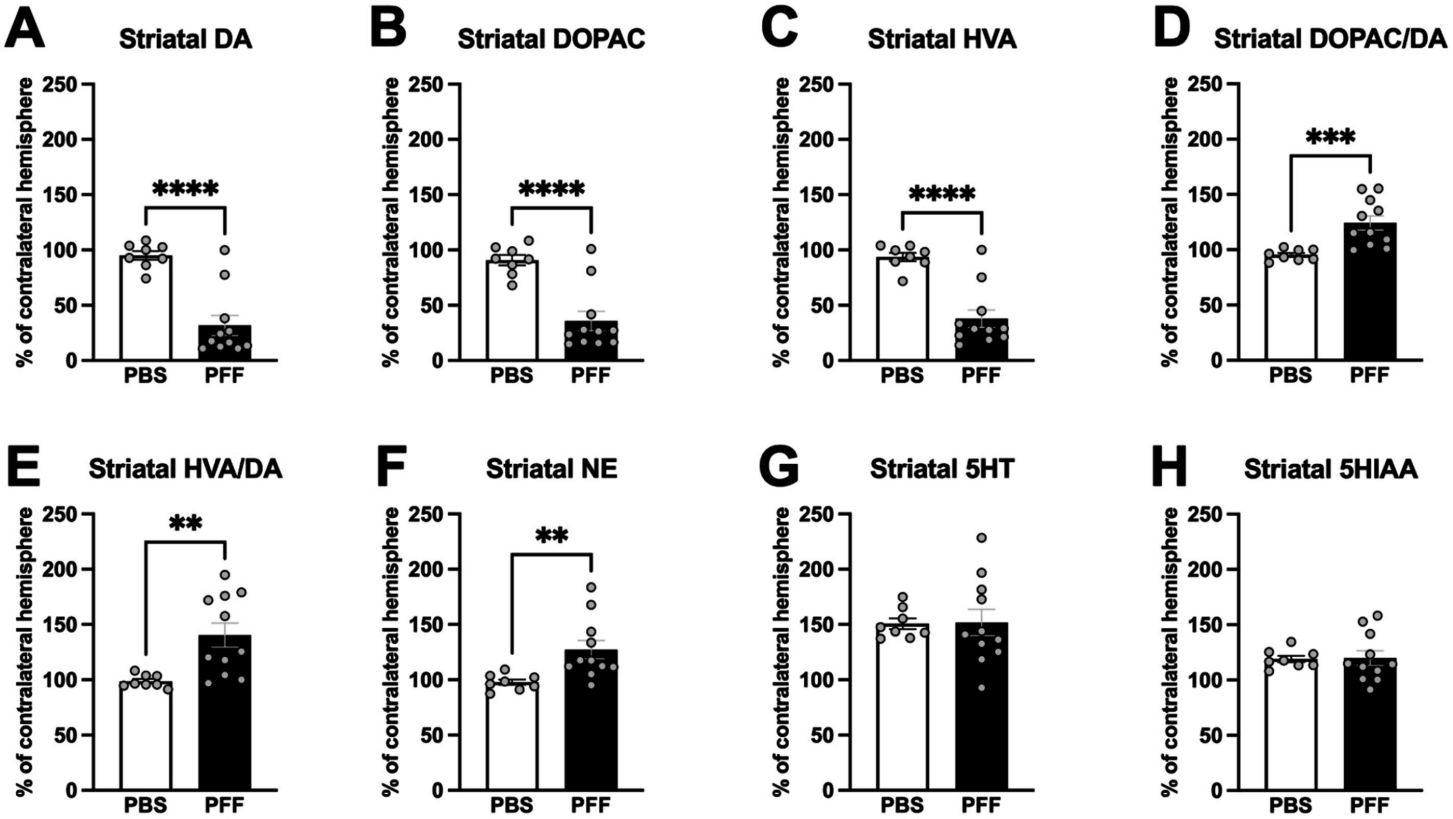
*α*-syn PFF injections are associated with decreased striatal dopamine and metabolites as well as increased DA turnover. HPLC analysis of striatal tissue content in PBS (*n* = 8) and PFF (*n* = 11) rats 8 months following surgery. Data is depicted as percent of ipsilateral striatal hemisphere relative to the contralateral striatal hemisphere. **(A–C)** Dopamine and dopamine metabolites in the striatum in PBS- and *α*-syn PFF-injected rats. **(D,E)** Dopamine turnover in the striatum of PBS- and *α*-syn PFF-injected rats **(F–H)** Norepinephrine, serotonin, and the serotonin metabolite 5HIAA were also measured in the striatum. Student’s *t*-test; **, **, **** *p* < 0.01, 0.001, and 0.0001, respectively.

Monoamine and metabolite levels were assessed in striatal and cortical tissue punches 8 months following intrastriatal *α*-syn PFF injection. Levels of all monoamines were relatively low in the cortex with no significant differences observed between surgical treatment groups and hemispheres (*p* > 0.05). In contrast, *α*-syn PFF injection was associated with significant reductions in DA, DOPAC, and HVA in the striatum, increased DA turnover and increased NE (Figure 8). Striatal DA levels in ipsilateral hemisphere of *α*-syn PFF rats were reduced ~70% compared to the contralateral hemisphere (Figure 8A; t(17) = 5.699, *p* < 0.0001), whereas DA levels in the striatum of PBS-injected were unchanged (Figure 8A, *p* > 0.05). DOPAC and HVA levels were similarly reduced in *α*-syn PFF injected rats (Figures 8B,C; DOPAC = t(17) = 5.017, *p* < 0.0001; HVA = t(17) = 5.605, *p* < 0.0001). DA turnover was increased in the *α*-syn PFF injected hemisphere, with both the ratio of DOPAC to DA (Figure 8D; t(17) = 3.791, *p* < 0.001) and HVA to DA (Figure 8E; t(17) = 3.220, *p* < 0.01) were increased. Interestingly, levels of NE in the striatum of *α*-syn PFF injected rats were significantly increased (Figure 8F; t(17) = 2.943, *p* < 0.01). Lastly, no significant differences in striatal 5-HT or 5-HIAA were observed between PFF and control rats (Figures 8G,H; *p* > 0.05).

### Motor and nonmotor performance are associated with striatal DA levels

**Figure 9:**
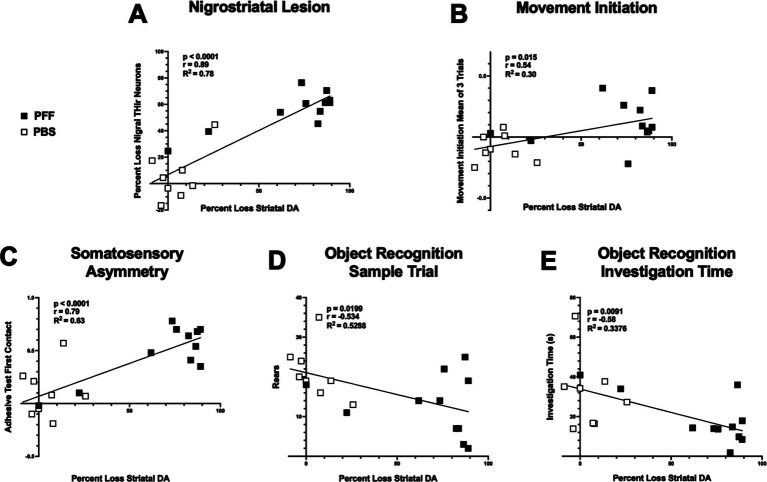
Motor and nonmotor performance are associated with striatal DA levels. Correlation analyses between striatal DA and THir and behavior in PBS and *α*-syn PFF rats. White squares represent individual PBS-injected rats, black squares represent individual *α*-syn PFF-injected rats. Loss of striatal DA tissue content is significantly associated with loss of nigral THir neurons **(A)**. Loss of striatal DA is associated with deficits in sensorimotor function including **(B)** movement initiation and (B) adhesive contact time. **(C)**. Loss of striatal DA is associated with both decreased rears **(D)** and decreased investigation time **(E)** in the object recognition test.

Lastly, correlation analysis was performed between nigral THir neuron loss and striatal DA levels within individual rats, as well as the relationship between striatal DA levels and movement initiation, somatosensory asymmetry, hindlimb stepping in the cylinder, and investigation time in the novel object recognition test (Figure 9). Not surprisingly, there was a strong positive correlation between the magnitude of loss of nigral THir neurons and the magnitude of striatal DA depletion (Figure 9A; *p* < 0.0001, *r* = 0.89, *R*^2^ = 0.78). Significant relationships also existed between striatal DA levels and the mean of three trials in the movement initiation test (Figure 9B; *p* < 0.05, *r* = 0.54, *R*^2^ = 0.30). Similarly, a significant correlation was observed between striatal DA levels and somatosensory asymmetry (Figure 9C; *p* < 0.0001, *r* = 0.79, *R*^2^ = 0.63). No relationship existed between striatal DA levels and hindlimb stepping in the cylinder (p < 0.05). More unexpectedly, there was a significant relationship between striatal DA levels and rears in the novel object recognition test (Figure 9D; *p* < 0.05, *r* = −0.534, *R*^2^ = 0.53) as well as with investigation time (Figure 9E; *p* < 0.01, *r* = −0.58, *R*^2^ = 0.34). An overall summary of the behavioral results and correlations are presented in Table 2.

**Table 2:** *α*-syn PFF-induced deficits, L-DOPA reversibility, and correlation with striatal DA summary.

Behavior	*α*-syn PFF-induced deficit	L-DOPA reversible	Striatal DA correlation
Movement initiation			
Mean of 3 trials	Yes	Yes	Yes
Somatosensory asymmetry			
Contact	Yes	No	Yes
Spontaneous activity			
Hindlimb steps	Yes	Yes	No
Object recognition			
Rears	Yes	NA	Yes
Investigation time	Yes	NA	Yes

NA, not assessed.

## Discussion

The *α*-syn PFF model has quickly become one of the most frequently utilized models in PD basic research. Its strengths include the development of endogenous *α*-syn pathology within neural circuitry connected to the injection site, followed by progressive nigrostriatal degeneration (Luk et al., 2012a; Duffy et al., 2018; Patterson et al., 2019a; Stoll et al., 2024a,b). In this study, the effect of unilateral intrastriatal injection of *α*-syn PFFs on sensorimotor and non-motor function in rats was investigated. The amount of *α*-syn PFFs and number of injection sites was increased relative to parameters used in our previous rat PFF studies in order to enhance seeding of SNpc neurons with pSyn inclusions, leading to greater nigrostriatal degeneration (Paumier et al., 2015; Duffy et al., 2018; Patterson et al., 2019a, 2024; Howe et al., 2021; Miller et al., 2021; Sossi et al., 2022; Stoll et al., 2024a,b; Centner et al., 2024). Results show that rats receiving *α*-syn PFFs develop significant impairments 6 months post injection in movement initiation, somatosensory asymmetry, hindlimb stepping in the cylinder, and object recognition. Furthermore, administration of L-DOPA improved movement initiation and increased hindlimb use in the cylinder. Post-mortem analysis of brain tissue showed accumulation of *α*-syn pathology and 55% loss of ipsilateral nigral DA neurons compared with PBS controls.

*α*-syn PFF rats displayed sensorimotor deficits in multiple motor tests at 6 months post-injection compared with controls. *α*-syn PFF injected rats were slower to initiate a step and to contact an adhesive stimulus with their contralateral limb compared with their ipsilateral limb. A modest reduction in hindlimb stepping in the cylinder was also observed in the *α*-syn PFF rats. These same tests have revealed contralateral impairments in the unilateral 6-OHDA rat model of PD with both severe and partial DA nigrostriatal lesions, highlighting their sensitivity and reliability (Kirik et al., 1998; Schallert and Tillerson, 2000; Fleming et al., 2005). The movement initiation test is particularly sensitive to striatal dysfunction and was originally designed by Schallert et al. (1992). It was initially used by Schallert and others to measure weight-bearing contralateral forelimb movements in the unilateral 6-OHDA rat model with severe nigrostriatal lesions (Schallert et al., 1992; Olsson et al., 1995; Tillerson et al., 2001). With a severe lesion, rats typically do not initiate any movement with the contralateral forelimb and thus, the test was considered an indicator of akinesia, a behavior at that time that was not reliably measured. However, as experiments using partial lesions became more common, the test was also shown to be sensitive to less severe DA lesions. With a partial lesion, rats will initiate a step, they are just slower to do so and thus, in this case the test is more a measure of bradykinesia or hypokinesia rather than a complete lack of movement (Schallert and Tillerson, 2000).

While the movement initiation test is primarily sensitive to striatal dysfunction, the somatosensory asymmetry test is sensitive to both cortical and striatal dysfunction (Schallert et al., 1982, 1983; Schallert and Whishaw, 1984; Barth et al., 1990; Schallert et al., 2000). In rats with unilateral 6-OHDA lesions or unilateral lesions of the forelimb sensorimotor cortex, bilateral stimulation of the radial/ulnar portion of the forelimbs results in ipsilateral limb preference in stimulus contact and removal. However, only in nigrostriatal DA lesioned rats is there also increased reactivity with the ipsilateral response, where rats make contact and remove the ipsilateral stimulus faster than sham controls (Schallert et al., 1982). In *α*-syn PFF rats, there was an increase in time to contact and remove the stimulus from the contralateral limb as well as an increase in ipsilateral reactivity. Ipsilateral contact times occurred within a mean of ~10 s for controls and ~ four seconds for *α*-syn PFF rats. Importantly, the contact and removal asymmetry in PFF rats is likely not due solely to nigrostriatal degeneration as cortical a-syn pathology is also well established in this model (Patterson et al., 2019a).

Hindlimb stepping in the cylinder was also modestly reduced in *α*-syn PFF rats at the 6 month timepoint compared to controls. Although earlier studies in the 6-OHDA rats focused mainly on forelimb use, the unilateral 6-OHDA mouse also show deficits in hindlimb stepping in the cylinder (Glajch et al., 2012). However, there are notable differences between the *α*-syn PFF rat and 6-OHDA mouse. The *α*-syn PFF rats stepped less with both contralateral and ipsilateral hindlimbs, while in the 6-OHDA mice the nigrostriatal lesion was severe and affected mice showed an asymmetry in stepping with the contralateral hindlimb making fewer steps.

Not all of the motor tests performed revealed behavioral deficits. No difference in contralateral stepping was observed in the adjusting step test in *α*-syn PFF rats in this study. This is somewhat surprising since we have previously observed deficits using a modified version of this test in *α*-syn PFF injected rats in which a slightly lesser magnitude of SNpc nigral loss (~51%) was observed, compared to the present study (Patterson et al., 2019a). In that study a moving treadmill belt was used to force stepping, while in the current study the rat was moved laterally across a surface by the experimenter. Thus, this difference in procedure may account for the different outcomes. More analysis in the treadmill version of the adjusting step test is warranted to compare the sensitivity of the two methods.

To investigate whether striatal DA depletion was contributing to the sensorimotor deficits observed in the *α*-syn PFF rats, saline and L-DOPA were administered followed by behavioral testing on movement initiation, somatosensory asymmetry, and spontaneous activity. Here, L-DOPA significantly reversed the movement initiation deficit and marginally improved hindlimb stepping in the cylinder. Improvement in both tests with L-DOPA has previously been reported in the unilateral 6-OHDA rat model (Olsson et al., 1995; Fleming et al., 2005). However, L-DOPA did not reverse the somatosensory asymmetry deficit. While it is not clear why somatosensory asymmetry was not altered by L-DOPA in *α*-syn PFF rats, as mentioned above, cortical lesions in the somatosensory cortex also produce somatosensory asymmetries and therefore, *α*-syn pathology in the cortex may significantly contribute to the observed deficit (Barth et al., 1990; Schallert et al., 2000).

Intrastriatal injection of *α*-syn PFFs results not only in nigrostriatal degeneration but also *α*-syn pathology in multiple extranigral connected brain regions including cortex, amygdala, and the anterior olfactory nucleus (Patterson et al., 2019a). Therefore, multiple non-motor tests were also performed that measure aspects of cognitive function (object recognition test), emotional reactivity (elevated plus maze), olfaction (block test), and sleep. In PD, impairments in multiple domains of cognition can develop, but deficits in executive function, attention, and visual spatial memory are most common, particularly in the early and mid-stages of the disease (Canavan et al., 1989; Owen et al., 1992; Levin and Katzen, 2005). In the *α*-syn PFF rats in this study, decreased attention was observed in an object recognition task. Six months following unilateral intrastriatal injection of *α*-syn PFFs, rats displayed a significant decrease in exploratory rearing and reduced investigation of objects compared with control rats. These data suggest an impairment in cognitive function since rearing was not affected in the cylinder test and there was no enhanced fear detected in the elevated plus maze in *α*-syn PFF rats, ruling out potential motor and emotional influences (Brown et al., 2010). Discrimination scores in object recognition did not significantly differ between groups and suggests that recognition memory is similar between PBS and *α*-syn PFF rats, but further cognitive testing is needed to determine whether multiple cognitive domains are impacted by *α*-syn pathology and neurodegeneration in this model. While cognitive function has not been frequently tested in the unilateral intrastriatal *α*-syn PFF rat model, studies have used *α*-syn PFFs to examine the effect on cognition. Closely related to the present study Marino et al. (2023) used bilateral intrastriatal injections of *α*-syn PFFs in rats and showed an impairment in investigation of a displaced object in a spatial version of the object recognition test. Other studies using *α*-syn PFFs to examine cognition have injected PFFs into the cortex and hippocampus or combined *α*-syn PFFs with viral vector overexpression of *α*-syn (Espa et al., 2019; Boutros et al., 2021; Zhang et al., 2023; Weber et al., 2024). These studies identified varying effects on working memory, aspects of behavioral flexibility, and spatial memory (Espa et al., 2019; Boutros et al., 2021; Zhang et al., 2023; Weber et al., 2024).

No differences were detected between PBS and *α*-syn PFF rats in the elevated plus maze, the block test of olfaction, or in sleep percent or bout length. However, only one test per non-motor behavior was assessed and potential effects on different aspects of these non-motor behaviors (e.g., REM sleep) cannot be ruled out.

Effect size (Table 3) and correlation analyses were performed to determine the strength of the differences between PBS and *α*-syn PFF groups and to compare extent of striatal DA lesion with the behavioral outcomes from tests in which *α*-syn PFF-associated impairments were observed. Effect sizes for the motor tests and object recognition ranged from medium to large indicating the strength of these tests in the *α*-syn PFF rat model. Movement initiation, somatosensory asymmetry, and aspects of object recognition were also significantly correlated with decreased striatal DA levels, providing further support that these specific behavioral assessments may be optimal for investigations in the *α*-syn PFF rat model.

**Table 3:** Effect size of behavioral outcomes.

Behavioral test	Cohen’s d	Pearson r
Movement initiation	1.41	0.57
Somatosensory asymmetry	1.52	0.60
Hindlimb steps in cylinder	0.89	0.41
OR- rears	1.17	0.51
OR- investigation time	0.81	0.38

OR, object recognition.

There are some limitations of the present study. All rats (PBS and *α*-syn PFF) received unilateral injections that passed through the cortex and into the striatum suggesting cortical damage incurred by the injection alone may also be a contributing factor. This is supported by behavior in the somatosensory asymmetry test observed in controls, although contact times between the ipsilateral and contralateral forelimbs were not statistically different, controls did show a tendency to contact the ipsilateral limb first. In addition, due to the sleep analysis conducted, there was a delay of ~6 weeks between sensorimotor/cognitive tests and postmortem analysis. During this interval it is possible that nigrostriatal degeneration progressed, albeit modestly (Centner et al., 2024). This would suggest that the sensorimotor and attention deficits we observed may be detectable at slightly lower magnitudes of nigral neuron loss, striatal denervation and striatal dopamine depletion than what we report in the present study. Lastly, whereas we did not observe sleep deficits following unilateral *α*-syn PFF injection, this may be due to lack of *α*-syn pathology within key ascending arousal system circuitry following intrastriatal PFF injection (Schwartz and Roth, 2008). Future studies that target PFF-induced synucleinopathy to arousal system circuitry are warranted.

## Conclusion

Research using the unilateral 6-OHDA rat model of PD led to the development of multiple sensitive and reliable sensorimotor tests that can detect movement impairments related to varying degrees nigrostriatal degeneration, examples include tests for spontaneous limb use (cylinder test), movement initiation, somatosensory asymmetry, and adjusting steps (Schallert and Tillerson, 2000). As animal models of PD continue to improve in their face validity, displaying both the *α*-syn pathology and nigrostriatal degeneration features of the disease, it is critical to determine whether PD-associated motor and non-motor deficits also are reproducible and reliable features of these models. Using the *α*-syn PFF model parameters described in the present study, resulting in augmented nigrostriatal degeneration, we demonstrate L-DOPA reversible sensorimotor deficits as well as deficits in attention. These same rat *α*-syn PFF model parameters should therefore be well-suited for testing the potential of novel therapeutics to mitigate symptoms that impact and reduce the quality of life for PD patients.

## Data Availability

The raw data supporting the conclusions of this article will be made available by the authors, without undue reservation.
